# Developing a Parenting App to Support Young Children’s Socioemotional and Cognitive Development in Culturally Diverse Low- and Middle-Income Countries: Protocol for a Co-design Study

**DOI:** 10.2196/39225

**Published:** 2022-10-31

**Authors:** Haley M LaMonica, Jacob J Crouse, Yun J C Song, Mafruha Alam, Mahalakshmi Ekambareshwar, Victoria Loblay, Adam Yoon, Grace Cha, Chloe Wilson, Madelaine Sweeney-Nash, Nathanael Foo, Melissa Teo, Mikael Perhirin, Jakelin Troy, Ian B Hickie

**Affiliations:** 1 Brain and Mind Centre The University of Sydney Sydney Australia; 2 Menzies Centre for Health Policy and Economics Sydney School of Public Health The University of Sydney Sydney Australia; 3 Minderoo Foundation Perth Australia; 4 BBE Melbourne Australia; 5 Faculty of Arts and Social Sciences The University of Sydney Sydney Australia

**Keywords:** child development, digital technology, global health, co-design, participatory research, stakeholder participation, mobile app

## Abstract

**Background:**

Digital technologies are widely recognized for their equalizing effect, improving access to affordable health care regardless of gender, ethnicity, socioeconomic status, or geographic region. The Thrive by Five app is designed to promote positive interactions between children and their parents, extended family, and trusted members of the community to support socioemotional and cognitive development in the first 5 years of life and to strengthen connections to culture and community.

**Objective:**

This paper aims to describe the iterative co-design process that underpins the development and refinement of Thrive by Five’s features, functions, and content. Minderoo Foundation commissioned this work as a quality improvement activity to support an engaging user experience and inform the development of culturally appropriate and relevant content for parents and caregivers in each country where the app is implemented.

**Methods:**

The app content, referred to as Collective Actions, comprises “The Why,” that presents scientific principles that underpin socioemotional and cognitive development in early childhood. The scientific information is coupled with childrearing activities for parents, extended family, and members of the community to engage in with the children to support their healthy development and to promote positive connections between parents, families, and communities and these young children. Importantly, the initial content is designed and iteratively refined in collaboration with a subject matter expert group from each country (ie, alpha testing). This content is then configured into the app (either a beta version or localized version) for testing (ie, beta testing) by local parents and caregivers as well as experts who are invited to provide their feedback and suggestions for improvements in app content, features, and functions via a brief web-based survey and a series of co-design workshops. The quantitative survey data will be analyzed using descriptive statistics, whereas the analysis of qualitative data from the workshops will follow established thematic techniques.

**Results:**

To date, the co-design protocol has been completed with subject matter experts, parents, and caregivers from 9 countries, with the first results expected to be published by early 2023. The protocol will be implemented serially in the remaining 21 countries.

**Conclusions:**

Mobile technologies are the primary means of internet connection in many countries worldwide, which underscores the potential for mobile health programs to improve access to valuable, evidence-based, and previously unavailable parenting information. However, for maximum impact, it is critically important to ensure that mobile health programs are designed in collaboration with the target audience to support the alignment of content with parents’ cultural values and traditions and its relevance to their needs and circumstances.

**International Registered Report Identifier (IRRID):**

DERR1-10.2196/39225

## Introduction

### Digital Health Solutions

In 2015, the United Nations General Assembly unanimously adopted the 2030 Agenda for Sustainable Development, which specifically highlighted the opportunity to capitalize on the spread of information and communications technology (ICT) to accelerate human progress worldwide [1]. Before and since that time, digital technology has acted as a revolutionizing force across diverse industries, and increasingly so in relation to health. Digital tools can directly influence some of the cultural and societal barriers to optimal health and development, such as accessibility and affordability. The Review Committee on the Functioning of the International Health Regulations (2005) during the COVID-19 response highlighted that strengthening IT systems and digitizing health systems are important ways of responding to and solving significant health challenges, even in developing countries [2]. Digitization of activities for parents and caregivers to promote childhood development is one such innovation [3]. For example, app-based digital technologies can also be used to provide information, maintain child development records (eg, vaccination records), and enhance access points to local and more distant health services.

### Technology Use in Low- and Middle-Income Countries

In recent years, there has been considerable growth in telecommunications infrastructure in low- and middle-income countries (LMICs). Table 1 highlights examples of large-scale initiatives in a range of LMICs that have driven ICT infrastructure growth to increase access to “smart” technologies to improve the standard of living. Despite such projects, it is estimated that 53% of people do not have access to the internet in developing countries, which stands in stark contrast to the 13% without access in developed countries [4]. Mobile technologies are the primary point of access to the internet in LMICs, with mobile broadband connections comprising 87% of total broadband connections [5]. However, gender remains a critical factor impacting access to mobile devices and, in turn, the internet. In LMICs, more than 300 million fewer women have access to the internet via a mobile device relative to men, with the widest gap in access evident in South Asia [6]. This reflects the way that technology use is mediated not only by access but by cultural practices and community norms [7]. Despite the marked digital divide, both men and women from LMICs report that ownership of a mobile phone coupled with internet access improves safety and facilitates access to previously unavailable information to improve daily life [6].

**Table 1:** Examples of technology infrastructure projects in low- and middle-income countries.

Country	Project
Afghanistan	A recent project funded by a US $50 million International Development Association Grant (Emergency Recovery Loan) resulted in a significant expansion in connectivity, increasing internet users from 15,000 in 2011 to 3.5 million in 2017 [8].
Indonesia	The Palapa Ring Project was recently completed in Indonesia, improving 4G internet access in all Indonesian cities as well as the regencies [9].
Kenya	Kenya’s “Vision 2030” includes plans to significantly upgrade the ICT^a^ infrastructure, including the expansion of fiber optic networks to public service institutions and providing 4G networks to ensure faster internet and improved bandwidth capacity [10].
Kyrgyzstan	In 2019, the Kyrgyzstan government adopted the “Digital Kyrgyzstan 2019-2023” strategy, which aims to improve digital infrastructure and access to the internet nationally, increase the digital literacy of its citizens through education, and develop e-government services [11].
Namibia	Namibia’s “Harambee Prosperity Plan II 2021-2025” includes the expansion of ICT infrastructure as one of its primary goals, with one aim being the provision of universal broadband access by 2025 [12].
Uzbekistan	In 2019, the Uzbekistan government approved the “Smart Cities” plan that aims to implement over 400 IT systems and infrastructure in the field of transportation, health, housing, education, and municipal services [13].

^a^ICT: information and communications technology.

In 2019, more than 204 billion apps were downloaded globally, reflecting an increase of approximately 5% compared with 2018 [14]. Social media apps, such as TikTok (ByteDance) and WhatsApp and Facebook (Meta), are consistently the most downloaded apps globally [15]. However, apps are being increasingly developed to address health-related problems in developing countries. For example, SASAdoctor, an app available via Android devices to all Kenyans, aims to improve the accessibility of affordable health consultations, particularly for the uninsured [16]. Furthermore, there is evidence of growing interest in and funding for mobile health (mHealth) programs to educate and empower women [17,18]. With the growth in the ICT sector generally and mobile phone subscriptions specifically, there is now a tremendous opportunity to implement mHealth initiatives globally and particularly in LMICs.

### Taking Account of Cultural Context

Mobile phones have become a powerful medium for disseminating important health messages. However, when considering mHealth initiatives, it is critical to understand the context, culture, attitudes, behaviors, and expectations of those for whom the digital solution is being designed [7]. With regard to child development, local culture significantly influences child care and parenting behaviors [19]. Furthermore, although parenting programs have generally been shown to be effective in improving children’s behavior [20], the same benefits are typically not evident in LMICs [21]. It has been suggested that cultural and linguistic differences and variability in accessibility and method of delivery may limit the usability and acceptability of such programs [22]. Failing to adequately account for context in population health interventions has been shown to result in ineffectiveness and has the potential to cause harm, widening health disparities by excluding access by some groups (eg, on the basis of ethnicity, language, or socioeconomic status) [23]. Although some mHealth interventions claim to be “universal,” a critical review highlights that their content is often underpinned by Western or individualist conceptualizations of the self, failing to incorporate non-Western or Indigenous perspectives on health and well-being [24]. Indeed, a meta-analysis showed that culturally adapted interventions were more effective than unadapted versions of the same intervention [25]. In addition, effective multicultural parenting programs have been shown to include culturally relevant content and provide information and skills training to bolster the confidence of parents [26]. Taken together, these findings underscore the critical importance of understanding the cultural values and traditions that drive parenting behaviors and parent-child and family-child interactions, including variations based on demographics, religion, and region. Accounting for cultural context will inform the iterative development and refinement of a parenting app, enabling it to be more appropriate and effective for the target audiences.

### The Importance of Co-design Methodologies

The World Health Organization’s Global Strategy on Digital Health (2020-2025) prioritizes the development and adoption of appropriate, acceptable, and scalable digital health solutions to promote health and well-being on a global scale [27]. Using strategies to enhance community and consumer engagement with digital health solutions is a priority in the health, medical, and research sectors internationally [28,29]. To that end, the Principles of Digital Development, which were created in consultation with a variety of international organizations including the Bill and Melinda Gates Foundation, the Swedish International Development Agency, the United Nations International Children's Emergency Fund, the United Nations Development Program, the World Bank, the US Agency for International Development, and the World Health Organization, highlight the importance of designing products in collaboration with the end user [30]. Co-design methodologies are essential for successful digital health projects, as they are an effective way to understand the context, culture, attitudes, behaviors, and expectations of those for whom the digital solution is being designed. In turn, this enables the solution to be adapted and iteratively refined to align with the local cultural context. Research has consistently shown that the active participation of all stakeholders (ie, consumers, researchers, product designers, etc) throughout the design of technical systems and services helps support the development of an end product that meets the needs of its intended user base, improves usability, and increases the engagement of all individuals [31,32]. Through the co-design process, end users become active partners in the development process, including idea generation, prototyping, and iterative feedback. In this way, co-design with end users promotes collaboration among research, industry, and local communities to address real-world problems [33,34].

### Thrive by Five

Minderoo Foundation is an Australian philanthropic organization whose vision for the Thrive by Five International Program is to inspire an increased understanding of and focus on the importance of early childhood development [35]. To facilitate this mission, the international Thrive by Five app, which will be free to use, is being developed for use by parents and caregivers in at least 30 countries around the globe, including countries in Africa (eg, Kenya, Namibia, and Ethiopia), Central Asia (eg, Kyrgyzstan and Uzbekistan), Southeast Asia (eg, Indonesia and Malaysia), South Asia (eg, Afghanistan), Middle East (eg, Jordan), North America (eg, the United States), and South America (eg, Brazil and Peru). In addition to increasing awareness of early childhood as a critical developmental period, the objectives of the Thrive by Five app are to (1) empower parents with the knowledge they need to support the healthy development of their child; (2) ensure universal access to this valuable parenting information regardless of region, socioeconomic status, literacy, gender, or other barriers; and (3) develop strong partnerships with in-country organizations to validate the cultural appropriateness and relevance of the app content as well as its features and functionality. Despite notable gains with regard to infrastructure development, limitations in access to devices, the internet, and electricity in many of the countries involved in this project may prevent some parents and caregivers from accessing the Thrive by Five app. Acknowledging that the app is the flagship of the broader Thrive by Five International Program, alternate means of disseminating content (eg, medical centers and hospitals, print media, radio, television, and WhatsApp) will be explored. The focus of this quality improvement research protocol is on the co-design, development, and refinement of the features, functions, and content of the app; however, the content developed through this process will be available for dissemination through a broader multichannel approach if necessary.

Minderoo Foundation contracted BBE, an Australian software development company, to design and develop the app’s features and functionalities. To complement this work, researchers from the University of Sydney partnered with Minderoo Foundation to support the development of the Thrive by Five content. Importantly, the content is underpinned by a scientific framework that highlights key neurobiological systems that support a child’s socioemotional and cognitive development (eg, reward, stress-response) and emphasizes a collectivist approach to parenting. The key elements of the content, referred to as “Collective Actions,“ are “The Why” that present scientific information about early childhood development in approachable, lay language and childrearing activities that parents, extended family, and the broader community can engage in with the child to support their development at different ages in the first 5 years of life.

This project will see the app implemented in 30 countries. Importantly, the content of the app will be adapted to the cultural context of each country to support its appropriateness and relevance. The aim is to celebrate the valued traditions of each country’s cultures while providing content to guide parents to best support their child’s development, given the challenges faced in each country. Critically, before embarking on work in any country, Minderoo Foundation identifies an in-country partner to provide support, guidance, and expertise throughout the duration of the project. This includes providing information about the local context; resourcing beta testers to test and review the app features, functions, and content; and assisting with app implementation and promotion. Over the course of the project, the partners are expected to be a mix of governmental and nongovernmental organizations with aims aligned with the Thrive by Five International Program, including the promotion of opportunities for healthy early childhood development and education.

### Objectives

By capitalizing on the current momentum in ICT infrastructure development and growing familiarity with and support for mHealth globally, we have the opportunity in this ambitious project to demonstrate the power of community engagement as a means to (1) develop and iteratively refine a novel mHealth app for parents; (2) facilitate the successful implementation and adoption of Thrive by Five to promote and optimize socioemotional and cognitive development of children from birth to age 5 years; and (3) strengthen relationships among parents, families, and communities with children in the early years and foster connections to community and culture. The objective of this paper is to outline the protocol for the co-design work that will be conducted as part of this project to support the development of an app that is usable, acceptable, and engaging and content that is culturally appropriate and relevant to the end users in each country. This work has been commissioned by Minderoo Foundation as a quality improvement activity to inform the iterative refinement of the Thrive by Five app.

## Methods

### Research and Development Cycle

#### Overview

**Figure 1:**
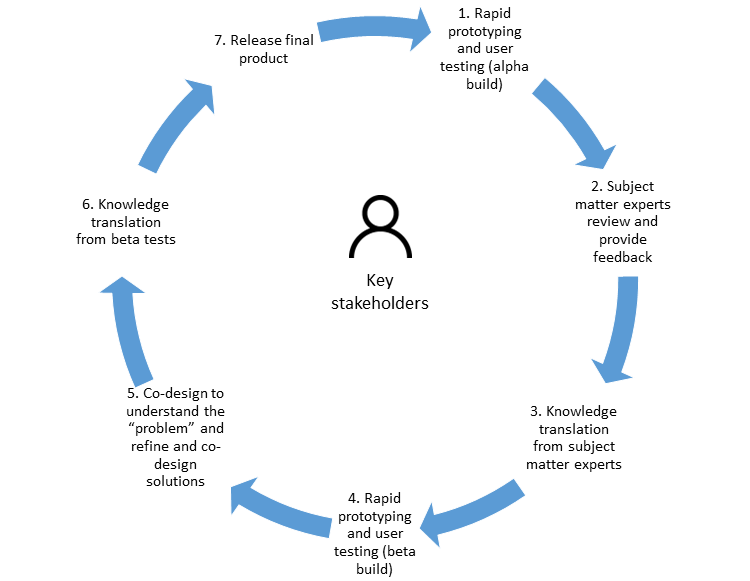
Research and development cycle.

The Medical Research Council’s Framework for Complex Interventions emphasizes an iterative approach to the development, feasibility testing, evaluation, and implementation of interventions [36]. The initial development phase focuses on stakeholder engagement and co-design to determine what outcomes might be expected from the development and implementation of the intervention in a real-world setting. In alignment with this approach, our research team’s established research and development (R&D) cycle, as illustrated in Figure 1, highlights the importance of co-design methodologies to explicitly position end users as empowered participants in all stages from design and development to implementation and evaluation [37-40]. This R&D cycle has been adapted to suit the needs of this large-scale international project, as explained in greater detail below. For ease of understanding, the alpha build refers to the first iteration of an app, whereas the beta build is a complete app that is used for testing to improve quality, usability, and user experience.

#### Alpha Testing

**Figure 2:**
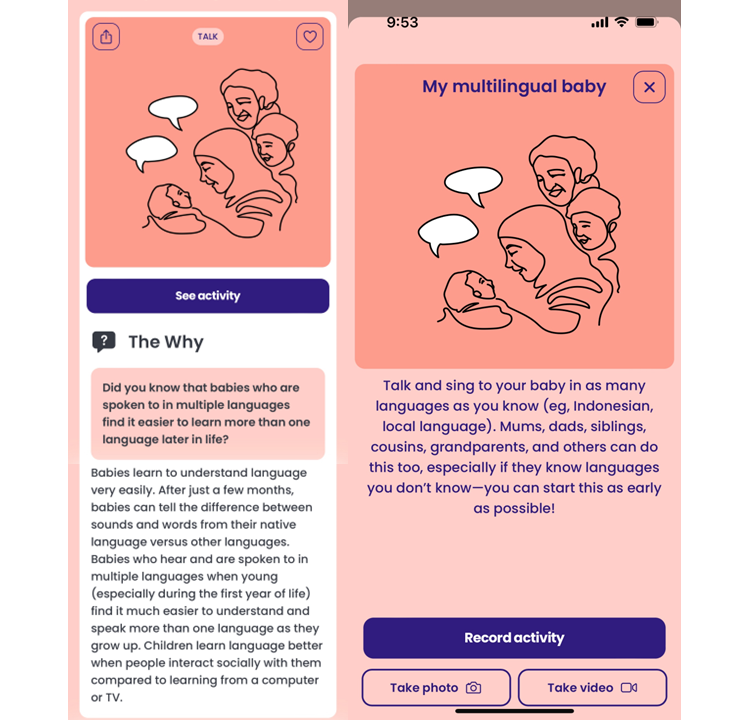
Example of a “Collective Action”.

The core features and functionality of the Thrive by Five app were established by BBE in partnership with Minderoo Foundation. Following a comprehensive literature review about the cultures, traditions, and values of the people and the history and social context of the target country, researchers from the University of Sydney develops preliminary content to populate the app, comprising 20-50 “The Why” and corresponding childrearing activities. As highlighted in Figure 2, the “The Why” reflects scientific information related to child development presented in lay language. This information is coupled with childrearing activities for parents, extended family, and trusted members of the community to engage in with the child to support their socioemotional and cognitive development. A subject matter expert (SME) group, convened by the in-country partner, is invited to provide feedback regarding the relevance and appropriateness of this initial content for the local context. The SME group may include, but is not limited to, specialists in early childhood development and education, psychology, medicine, and anthropology as well as representatives from relevant government ministries. The input of this group is critical to support the researchers in developing and tailoring content to the cultural context, supporting its relevance and applicability to the potential users. Having tested both the app functionality and content internally (ie, alpha testing), either a beta or a local version of the app is created for each country for testing by real-world users (ie, beta testing).

#### Beta Testing

##### Overview

A representative sample of parents and family members as well as key local stakeholders (eg, preprimary schoolteacher and child psychologist) will be invited to test Thrive by Five naturalistically (ie, in a manner of their own choosing) for a minimum of 1 week. Any participant incentives, such as transport, catering, and vouchers, will be determined by the in-country partner. In the first 2 countries (ie, Indonesia and Afghanistan) where the proof of concept for the Thrive by Five app was tested, more than 50 users were invited to test the app. For all future implementations, at least 25 beta testers will be invited to test the app. The identification of beta testers will be facilitated by the in-country partner. Testers will use the app at their own discretion during this period; no specifications will be set as to the frequency of use or number of activities completed. To ensure that a diversity of voices contribute to the testing process, whenever feasible, participants will be recruited from metropolitan, regional, and rural communities, including men and women from varied demographic backgrounds (eg, education, socioeconomic status, literacy, and religion). Although it will not always be feasible to include individuals from remote locations in the workshop activities, various strategies will be used to facilitate their engagement, including conducting workshops in regional areas, transporting participants from regional areas to workshop locations, using videoconferencing technologies, and allowing participants to provide written feedback and suggestions for content.

##### Quantitative Data

All testers will be asked to complete a brief questionnaire about their experience of using Thrive by Five and its impact on their feelings of connectedness to their child. The questions that will be asked to the beta testers are presented in Multimedia Appendix 1, with the corresponding response options.

In addition to direct quantitative user feedback, data analytics embedded in the Thrive by Five app or website will be available to examine user behavior (eg, frequency of use, average time of each use, and features with longest and shortest average periods of engagement) to determine which features are preferred relative to those that may require refinement or removal.

##### Qualitative Data

Central to the R&D cycle are co-design workshops. Direct engagement with potential end users through workshops is essential for testing and evaluating the user experience, functionality, and features. Furthermore, workshop participants will be asked to provide feedback on the relevance and cultural appropriateness of the content of the Thrive by Five app. All data from the workshops will be used to iteratively develop and refine the digital tool. Given the disparities in demographics (eg, age, education, and marital status), literacy, socioeconomic status, and cultural traditions and beliefs, workshop participants may be grouped based on commonalities in demographics and culture, in accordance with guidance from the in-country partner. This distribution of workshop participants is intended to enable all participants to feel comfortable in sharing their honest opinions, experiences, and beliefs.

**Figure 3:**
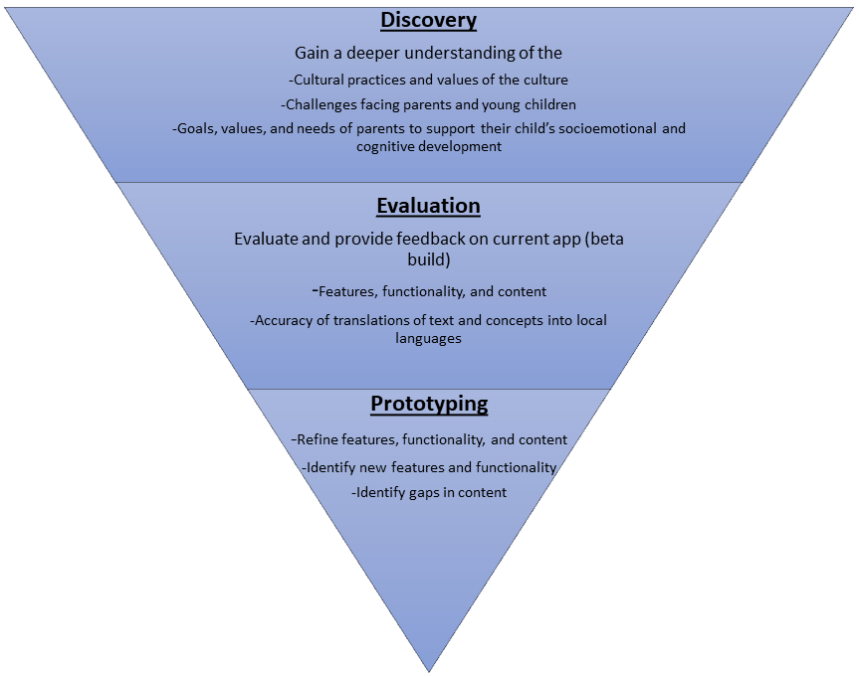
Structure of each co-design workshop.

Up to six 1.5- to 3-hour co-design workshops (depending on the need for translation), each with 4 to 6 participants, will be conducted via Zoom (or alternate video conferencing technology) over a 4-week period using an agenda consisting of 3 stages: “discovery,” “evaluation,” and “prototyping” (Figure 3). The workshop time will be halved, with the first part being used to explore expert, parent, and caregiver feedback on the app user experience, features, and functions, and the second part being dedicated to exploring how the content can be developed for and tailored to the needs of the parents and caregivers in each country. The workshops will be cofacilitated by representatives from the University of Sydney, Minderoo Foundation, and BBE in collaboration with a “local champion” identified by the in-country partner. With regard to the latter, the local champion does not need to have specialist credentials but rather a connection to the community and an eagerness to support the objectives of the Thrive by Five International Program. Research has shown that such champions can increase the awareness of and interest in participating in research among potential participants as well as other stakeholders involved in the research process (eg, health professionals) [41]. They will help ensure that all workshop participants feel comfortable sharing their open and honest feedback. Whenever possible, one or more facilitators will conduct the workshops in person, with other facilitators participating via video conferencing. Detailed notes will be taken by 2 scribes from the University of Sydney. In addition, the workshops will be recorded to ensure the accuracy of the notes. Depending on the language used to conduct the workshops, the in-country partner may be required to provide a translator to ensure that all participants are able to communicate in their preferred language. No formal certification is required to serve as a translator for this project, and in-country partners are responsible for identifying individuals with an appropriate level of fluency in the required languages. Simultaneous translation will be used whenever possible. If consecutive translation is required, facilitators and workshop participants will pause after every 1-3 sentences to enable accurate translations. Any questions arising from the translation will be checked with the in-country partner to ensure clarity.

A variety of methods will be used during the workshops to promote engagement from participants, including primarily prompted discussions and the development of user personas and specific use cases as appropriate. Importantly, BBE and Minderoo Foundation are responsible for leading the discussion regarding user experience, including the features and functionality. The researchers are responsible for leading the discussion about the cultural context into which the app and content are being implemented with the aim of developing content to promote and optimize socioemotional and cognitive development of children from birth to age 5 years. A sample agenda for a series of workshops for a country is available in Multimedia Appendix 2. Topics for discussion will be sent to participants at least 1 week before the workshop to allow time for preparation for those who choose to do so. The agenda questions are designed to identify challenges faced by parents and caregivers of children aged <5 years that may be able to be addressed through the Thrive by Five content. In addition, the questions explore traditional customs and beliefs that can be used to help contextualize app content.

The workshops support the exploration of a number of critical areas, such as the (1) look and feel of the app, including the design, language, tone of voice, colors, and illustrations; (2) usability, acceptability, and engagement of the Collective Actions; (3) relevance and appropriateness of the Collective Actions; (4) desired attributes, skills, and values for children; (5) information about children’s socioemotional and cognitive development that would be of benefit; (6) essential people (kin or other caregivers) in a child’s life who may benefit from shared use of the app to support childrearing; (5) barriers to and facilitators of the uptake and adoption of the app and the Collective Actions; (6) preferred features, functionality, and content; and (7) gaps in the test version, including features, functionality, and content. These research questions are critical to support the development of content for the final product that is appropriate for the cultural context. In addition, the co-design work will facilitate the discovery of areas for future development.

#### Refinement and Future Content Development

Learnings from the beta testing process will then be examined internally by the research team to identify content that requires revision and gaps in the content to be developed for the end product. Importantly, a full library (ie, at least 100) of “Collective Actions” will be developed before implementation, all of which will be validated by the SME group. The research team will also identify key themes (eg, health advice, information about childhood nutrition, and early childhood learning activities) to guide future content development, including the potential to create alternate forms of content, such as longer-form articles. Furthermore, the developers from BBE, in collaboration with the researchers and Minderoo Foundation, will review the experiences reported by beta testers in relation to the app features and functionality. This process enables the technology team to consider ways to refine the app, either before implementation or for future iterations. Over the course of this project, it is anticipated that new features, functions, and content will be developed, tested, and implemented as part of the Thrive by Five app based on cumulative learnings, thus benefiting future countries embarking on the co-design process as well as countries that have already implemented the app.

### Knowledge Translation and Qualitative Analysis

Knowledge translation refers to the synthesis, exchange, and application of knowledge by stakeholders to enhance the benefits of innovation in strengthening health systems and improving health outcomes [42]. The aim is to promote the translation of research findings into technology development and real-world implementation, bridging what has been coined “the know-do gap” [42]. In this instance, the knowledge translation process will inform the iterative refinement of the app to enhance its usability, acceptability, relevance, and cultural appropriateness for each of the 30 target countries.

Descriptive statistics will be used to characterize the sample of beta testers and to analyze the findings from the web-based surveys. Interpretation of the qualitative data from the workshops will follow established thematic techniques (ie, inductive reasoning) [43]. All raw data will be reviewed and checked across all participants by a senior researcher to develop a coding framework tailored to each country’s context, outlining all the key concepts. Subsequently, the data will be coded in NVivo 12 software (QSR International) using this framework by 2 researchers. The coding will follow an established iterative process of reading, coding, and exploring the pattern and content of coded data, followed by reflection and discussion to reach consensus.

### Ethics Approval

This study has been approved by the University of Sydney Human Research Ethics Committee (protocol 2021/956).

## Results

The co-design process has been completed in Indonesia, Afghanistan, Namibia, Kenya, Kyrgyzstan, Uzbekistan, the Democratic Republic of the Congo, Cameroon, and Ethiopia, with the results expected to be published in early 2023. This includes participation of a total of 174 parents and caregivers and 58 in-country SMEs in 55 workshops in the 9 countries between January 2021 and August 2022. The remaining 21 countries will commence work on a rolling basis. A report summarizing the qualitative and quantitative findings from each country will be provided to Minderoo Foundation to inform their quality improvement processes.

## Discussion

### The Value of Co-design in the Development of Digital Health Solutions

Co-design is recognized as a critical component in the development of digital health solutions, ensuring that the products meet the needs of end users. However, a well-designed product in isolation is not sufficient to drive improved outcomes. In this project, we place considerable emphasis on the cultural appropriateness of the content for local settings to inform the iterative development of the Thrive by Five app and content to support its acceptability and relevance in a range of countries globally. Importantly, a qualitative study conducted with 162 ethnically diverse North American parents (63% non-Hispanic White, 14% African American, 10% Hispanic, 9% Asian or Pacific Islander, and 4% other) found a preference for self-administered parenting programs, such as via television, web-based programs, or written materials, as opposed to those requiring home visits, therapists, and weekly groups [44]. To that end, apps are cost-effective and well placed to fit into parents’ daily routines. However, research has shown that standardized, evidence-based apps designed specifically to support research objectives, with no opportunity for iterative refinement, are far less popular than commercial apps among users [45].

Given the previously mentioned findings, to facilitate successful engagement and adoption, it is essential to capitalize on the adaptability of apps, including the potential to fix bugs and glitches and improve functionality to enhance user experience. However, and perhaps more importantly, co-design methodologies enable the iterative refinement of apps and will be used in this project to improve the cultural relevance and appropriateness of the Thrive by Five app by actively integrating local cultural practices into the Collective Actions to strengthen family and community relationships and foster connections to cultural traditions and values from a young age. Notably, a systematic review of 72 studies presenting evidence from studies of Indigenous communities globally found consistently positive associations between cultural connections and health and well-being outcomes [46]. Greater participation in culture has been shown to have positive effects on social and emotional well-being; physical health, including blood pressure, BMI, and cholesterol levels; and health-related lifestyle factors, such as smoking, alcohol consumption, nutrition, and physical activity levels [46]. These findings highlight the holistic nature of health and well-being and the importance taking into account cultural traditions and values when designing, developing, and implementing health-related digital health solutions. This is particularly important when engaging with communities and cultural groups not typically involved in more traditional research methods, such as clinical trials [47].

### Conclusions

Several programs have been developed to support early childhood education and reduce the loss of developmental potential for disadvantaged children in LMICs. A review of 63 studies of such programs highlighted three factors critical to improving outcomes for children: (1) guidance about activities to support the socioemotional, cognitive, and behavioral development of the child; (2) skills training for parents and other caregivers to enhance the cognitive stimulation of the child and to foster a supportive environment; and (3) emphasis on parental health, well-being, and self-efficacy. Importantly, these key elements are already being developed and incorporated into the beta versions of the Thrive by Five app. However, the vital co-design process will emphasize the validation of the cultural appropriateness and relevance of all content as well as provide an opportunity to identify areas for improvement, adaptation, and refinement for the unique context present in each country.

## Appendix

Multimedia Appendix 1 Survey questions and responses to collect feedback on the beta version of Thrive by Five.

Multimedia Appendix 2 Co-design workshop agenda.

## Abbreviations

ICT: information and communications technology
LMIC: low- and middle-income country
mHealth: mobile health
R&D: research and development
SME: subject matter expert
