# Supplementary material for: Developing a Parenting App to Support Young Children’s Socioemotional and Cognitive Development in Culturally Diverse Low- and Middle-Income Countries: Protocol for a Co-design Study
Source: JMIR Res Protoc. 2022 Oct 31;11(10):e39225. doi: 10.2196/39225 (PMC9664325; doi:10.2196/39225)
Supplement: Multimedia Appendix 1 [file resprot_v11i10e39225_app1.docx]

**Multimedia Appendix 1: Survey questions and responses to collect feedback on the beta version of Thrive by Five**

| Are you a mother or father of a child 0-5 years? | | | | | | |
| --- | --- | --- | --- | --- | --- | --- |
| Mother | | Father | | | Other | |
| If other, are you a caregiver or close relative or friend of a child between 0-5 years? | | | | | | |
| Caregiver | | Close relative or friend | | | Other | |
| How many times have you used Thrive by Five in the past week? | | | | | | |
| Free text numerical response to indicate the frequency of use in the last week | | | | | | |
| I learned new ways to support the child’s future from Thrive by Five. | | | | | | |
| Strongly disagree | Disagree | | Neutral | Agree | | Strongly agree |
| Using Thrive by Five over the past week has made me feel more connected to the child. | | | | | | |
| Strongly disagree | Disagree | | Neutral | Agree | | Strongly agree |
| Using Thrive by Five over the past week has made our family and relatives feel more connected to the child. | | | | | | |
| Strongly disagree | Disagree | | Neutral | Agree | | Strongly agree |
| The activities in Thrive by Five are appropriate for parents and families in [insert name of country]. | | | | | | |
| Strongly disagree | Disagree | | Neutral | Agree | | Strongly agree |
| Thrive by Five has improved my confidence as a parent. | | | | | | |
| Strongly disagree | Disagree | | Neutral | Agree | | Strongly agree |
| Would you recommend Thrive by Five to other parents or families? | | | | | | |
| No, I would not recommend this app to anyone | There are very few people I would recommend this app to | | Maybe, there are several people I would recommend this app to | There are many people I would recommend this app to | | Definitely, I would recommend this app to everyone |
| How many times to do you think you would use Thrive by Five in the next 12 months? | | | | | | |
| 0 | 1-2 | | 3-10 | 10-50 | | >50 |
| What other content or features would you like to see in the Thrive by Five app? | | | | | | |
| Free text response | | | | | | |
| How well did Thrive by Five work on your phone? | | | | | | |
| 0  App did not work | 1, 2, 3, 4 | | 5  A few small problems | 6, 7, 8, 9 | | 10  No problems at all |
| Overall, what score would you give Thrive by Five? | | | | | | |
| 0  Not good | 1, 2, 3, 4 | | 5  Neutral | 6, 7, 8, 9 | | 10  Excellent |
