# Supplementary material for: Developing a Parenting App to Support Young Children’s Socioemotional and Cognitive Development in Culturally Diverse Low- and Middle-Income Countries: Protocol for a Co-design Study
Source: JMIR Res Protoc. 2022 Oct 31;11(10):e39225. doi: 10.2196/39225 (PMC9664325; doi:10.2196/39225)
Supplement: Multimedia Appendix 2 [file resprot_v11i10e39225_app2.docx]

**Multimedia Appendix 2: Co-Design Workshop Agenda**

N.B. This is a list of questions that we will try to cover throughout the workshops for each country. Depending on the time and responses it is assumed that not all questions can be covered in one workshop. Like previous workshops the untouched questions/themes will be prioritized in the following workshops.

**Content specific questions:**

1. What do you think about the activities, information, pictures, and language in the app? Anything you liked or did not like?
2. (i) What information would parents and/or caregivers in [country] want from the app to help care for children (0-5 years)?

(ii)Follow up: Are there any specific challenges (regarding parenting and child development) that we might be able to address through the app?

1. When you think back to being a first time parent (depending on number of children), what questions did you have? What did you want to know about raising a child?
2. In your family, who will be using this information (and app) most often?
3. Would any group of parents find the content more useful than the other (e.g. urban vs rural, working mum vs stay-at-home mum, mums vs dads, single mums)?

**Child development specific questions:**

1. What values or morals do you want to instill in children?
2. How do children learn about their culture and traditions (e.g. costumes, festivals, stories, plays etc.)?
3. What skills do you want your child to learn by the age of 5?
4. (i) Could you tell us about how children under the age of 5 spend their time during the day and before going to bed (first ask about weekdays and then about weekends)?
5. Follow up (if not covered in the above question): Who looks after children during the day and before they go to bed?
6. (If not covered in the two questions above) What is the daily schedule of an [country] parent/caregiver on a weekday and on weekends?
7. How do young children socialize with other children in their community?
   1. Do children have favourite games or activities that they do together?
8. What is the role of television and mobile phones in children’s day-to-day activities?

**Parenting related questions:**

1. (i) How do you manage tantrums and emotions of young children?
2. Follow up- Is there any parenting strategy that you like or found useful?
3. Do you receive parenting information or help from any other sources?
4. Is there anything you want to know from us?

**Access issues**

1. What kind of technology or devices do you and your family use? How is your access to the internet?
2. Do you think any special training is required to use the app?
3. When do you think parents would make the best use of the app (morning/day/night, when they are with the kid/not without the kid, weekends)?
4. Are there any issues (electricity, lack of smart phone) that would prevent parents from using the app?
5. For people unable to access the app, how can we deliver the information to them?
